# Shock Acceleration and Attenuation during Running with Minimalist and Maximalist Shoes: A Time- and Frequency-Domain Analysis of Tibial Acceleration

**DOI:** 10.3390/bioengineering9070322

**Published:** 2022-07-16

**Authors:** Liangliang Xiang, Yaodong Gu, Ming Rong, Zixiang Gao, Tao Yang, Alan Wang, Vickie Shim, Justin Fernandez

**Affiliations:** 1 Faculty of Sports Science, Ningbo University, Ningbo 315211, China; lxia467@aucklanduni.ac.nz (L.X.); gaozixiang111@163.com (Z.G.); 1565409093@gmail.com (T.Y.); 2 Auckland Bioengineering Institute, The University of Auckland, Auckland 1010, New Zealand; alan.wang@auckland.ac.nz (A.W.); v.shim@auckland.ac.nz (V.S.); j.fernandez@auckland.ac.nz (J.F.); 3 Faculty of Engineering, University of Pannonia, H-8201 Veszprém, Hungary; 4 Faculty of Medical and Health Sciences, The University of Auckland, Auckland 1010, New Zealand; 5 Department of Engineering Science, Faculty of Engineering, The University of Auckland, Auckland 1010, New Zealand

**Keywords:** running, impact loading, shock acceleration, shock attenuation, minimalist shoes, maximalist shoes

## Abstract

Tibial shock attenuation is part of the mechanism that maintains human body stabilization during running. It is crucial to understand how shock characteristics transfer from the distal to proximal joint in the lower limb. This study aims to investigate the shock acceleration and attenuation among maximalist shoes (MAXs), minimalist shoes (MINs), and conventional running shoes (CONs) in time and frequency domains. Time-domain parameters included time to peak acceleration and peak resultant acceleration, and frequency-domain parameters contained lower (3–8 Hz) and higher (9–20 Hz) frequency power spectral density (PSD) and shock attenuation. Compared with CON and MAX conditions, MINs significantly increased the peak impact acceleration of the distal tibia (*p* = 0.01 and *p* < 0.01). Shock attenuation in the lower frequency depicted no difference but was greater in the MAXs in the higher frequency compared with the MIN condition (*p* < 0.01). MINs did not affect the tibial shock in both time and frequency domains at the proximal tibia. These findings may provide tibial shock information for choosing running shoes and preventing tibial stress injuries.

## 1. Introduction

Running is a prevalent worldwide form of exercise and with multiple benefits. However, runners suffer a high injury rate in their lower limbs [1,2]. Impact loading is a crucial measure to evaluate kinetic performance, and it is potentially associated with running-related injuries, such as patellofemoral pain and plantar fasciitis [3]. Ground reaction force (GRF) metrics, such as vertical peak GRF and vertical instantaneous load rate (VILR), are commonly employed to indicate impact loading during running. A greater VILR in runners has been associated with an increased risk of injury [3]. However, GRF metric measures usually need to be conducted in the laboratory setting with a force platform embedded in the running path or treadmill. Tibial acceleration has been shown to be strongly correlated with GRF metrics during running [4]. Moreover, collecting tibial acceleration from wearable inertial measurement unit (IMU) sensors is convenient outside the gait lab, cost-saving, and could increase the ecological validity.

Different running shoes are designed to decrease running-related lower limb injuries. Although modern shoes with cushioning are made to respect the natural foot shape and function evolutionarily, they may modify natural biomechanical characteristics during running [5]. Barefoot running has attracted lots of attention in the past decades. It can stimulate and strengthen inner foot muscles and maintain longitudinal arch function [6]. Inspired by barefoot running, minimalist shoes (MINs) aim to promote the natural movement of the foot and obtain barefoot-like biomechanical benefits during running, but without plantar surface injuries (i.e., blisters and bruises) [1]. MINs are characterized by a high flexibility, low weight, midsole stack height, and heel-to-toe drop without motion control and stability technologies/devices [5]. MIN running has been supported to promote foot function [7,8,9,10] and increase intrinsic foot muscle strength [7,11]. Nevertheless, rearfoot strike running in MINs may increase the impact loading rate, which has been suggested to increase the likelihood of injury in the shin and calf [12]. MIN running also presents a more pronounced instantaneous and average loading rate than conventional shoes (CONs) [13,14].

Maximalist shoes (MAXs) are distinguished by a high midsole stack height (typically greater than 30 mm) and excellent shock absorption properties [15,16]. They have been advertised in recent years with increased cushioning to protect runners from potential running-related injuries. Runners with MAXs may generate a smaller VILR than with MINs [12,13]. However, there are also some debates raised from recent studies. A previous study found that impact loading was increased after 5 km of running with MAXs [16]. MAXs may be unable to significantly decrease the impact loading metrics [17]. Nevertheless, limited studies have investigated tibial acceleration between MAXs and MINs [18,19,20].

Shock acceleration and attenuation characteristics in running may be affected by many factors. Peak tibial acceleration in the time and frequency domains was increased significantly for the habitual rearfoot runners than forefoot runners. Rearfoot runners also presented a significant shock attenuation effect in the lower and higher frequency ranges [21]. Lower limb impact attenuation is increased with the increase in step length during running [22]. Decreasing stride frequency resulted in a greater tibial impact acceleration and power spectral density (PSD) of the signal [23]. It was also found that peak tibial acceleration increased following a prolonged run, but shock attenuation did not change from a previous study [24]. During running, time- and frequency-domain features of the shock acceleration reflect the impact loading and attenuation functions of the footwear in the lower limbs. Sinclair et al. [13] demonstrated that runners exhibited higher tibial acceleration when wearing MINs than MAXs.

However, there is no compelling evidence supporting the differences in tibial shock acceleration in the time and frequency domains and how tibial shock attenuation changes between MAXs and MINs. Furthermore, recreational runners are frequently troubled by lower limb injuries, especially around the tibia, and the tibia absorbs a significant portion of the impact acceleration. Therefore, this study aimed to investigate the shock acceleration and attenuation in the tibia among MAXs, CONs, and MINs. We hypothesized that: (1) MINs increase and MAXs decrease peak shock compared to CONs in the time domain; (2) MIN running exhibits a significant shock attenuation effect due to it exhibiting a more natural barefoot running gait pattern from an evolutionary perspective and a greater power spectral magnitude at the distal tibia.

## 2. Materials and Methods

### 2.1. Participants

A minimum of twenty-one participants were required for this study (power: 0.8, effect size: 0.25, α = 0.05 and β = 0.2). Therefore, this study recruited twenty-four male recreational runners (age: 28.3 ± 1.1 years, height: 1.76 ± 0.04 m, mass: 65.8 ± 2.2 kg, BMI: 21.3 ± 0.3 kg/m^2^) from the university and local running clubs. Considering the differences in running mechanics between males and females [25], this study only focused on male participants. Inclusion criteria included recreational level runners, right leg-dominant runner, habitual rearfoot strike runners, and those who never had run in MINs or MAXs previously. The recreational runner was defined as running 2–4 times per week with a weekly running mileage no less than 20 km, and an age-graded score < 60th percentage calculated based on age, gender, and race performance in the past six months [26]. The rearfoot strike pattern was defined by the strike index (center of pressure within 0–33% of the foot length at the initial contact) using the Footscan^®^ pressure plate (Rsscan International, Olen, Belgium) [27]. The test was conducted while participants were wearing the CONs. Exclusion criteria were as follows: BMI out of the range of 18–25, neurological or cardiovascular diseases, pes planus or pes cavus, and lower limb musculoskeletal injuries within the six months prior to participation in this study. All participants were free to exit the experiment at any test stage, and written informed consent was obtained from each participant before the test. The study protocol was conducted in compliance with the declaration of Helsinki and was approved by the University’s Institutional Review Board (RAGH20201137).

### 2.2. Experiment Protocol

**Figure 1:**
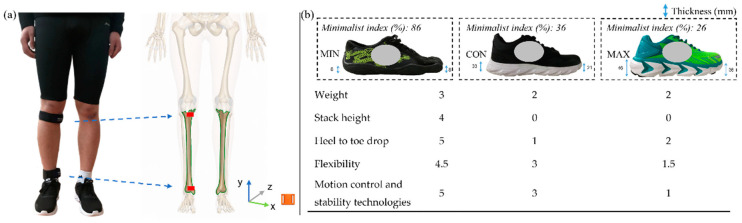
The sensor placement (**a**) and the minimalist index and sub-scores for each item (**b**). Note: MINs: the minimalist shoes; CONs: the conventional shoes; MAXs: the maximalist shoes.

Participants were instructed to maintain their foot strike pattern during the test to avoid the effects of foot strike pattern change on the findings. Each participant was given ten minutes to run on the treadmill (Quasar, h/p cosmos^®^, GmbH, Germany) at a speed of 8 km/h for a warm-up and to become familiarized with the different shoes and experimental setting. All runners were required to wear a short running garment during the test. Tri-axial accelerometers (IMeasureU V1, Auckland, New Zealand; 40 × 28 × 15 cm, weight: 12 g, resolution: 16 bit) were attached on the proximal and distal anteromedial tibia of each participant’s dominant leg using the strap, with the vertical axis aligning with the tibia (as shown in Figure 1a). All participants ran 6 min on the treadmill for each shoe condition with a speed of 10.8 ± 0.5 km/h, calculated according to the Froude velocity [28]. During the test, each subject ran 5 min to ensure that their gait stabilized and the last minute was record. The order for footwear selection was assigned randomly, and there was at least a ten-minute (10–30) break between each session to avoid fatigue effects [15,20]. The minimalist index was assessed among MINs, MAXs, and CONs, being 86%, 26%, and 36%, respectively (EURO sizes: 41–43); detailed information for each item is presented in Figure 1b. Each item was scored from 0 to 5. The minimalist index was evaluated based on an expert consensus from Esculier et al. [5] and was calculated by adding up all sub-scores, then multiplying by 0.04.

### 2.3. Data Collection and Processing

**Figure 2:**
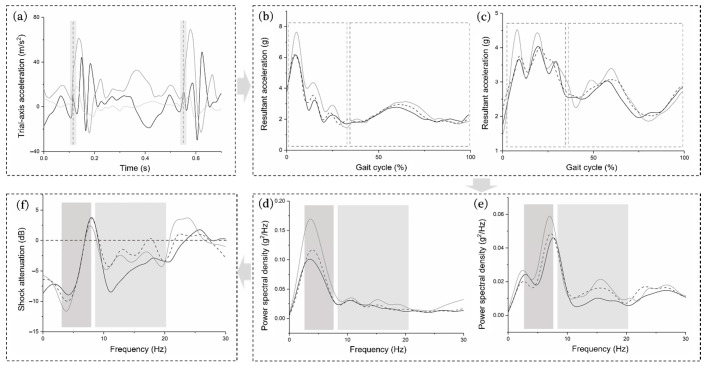
Graphical representation of the acceleration data process technique in the time and frequency domains. These include raw time-series tri-axis acceleration data (**a**); resultant acceleration from the distal (**b**) and proximal (**c**) tibia; power spectral density from the distal (**d**) and proximal (**e**) tibia; shock attenuation from the distal tibia to the proximal tibia (**f**). Note: black denotes maximalist shoes, gray denotes minimalist shoes, and the dashed line indicates conventional shoes.

The tri-axis acceleration signal (Figure 2a) was sampled at 500 Hz and was filtered using a second-order, low-pass, zero-lag Butterworth filter with a cutoff frequency of 60 Hz to remove noise based on spectral analysis. Resultant accelerations were calculated as $\sqrt{x^{2}+y^{2}+z^{2}}$ (Figure 2b,c). We picked four steady gait cycles from each 10 s of the oneminute acceleration data according to the previously established and validated method, whereas the initial foot contact was the local minima within the 75 ms prior to the peak resultant distal tibial acceleration [29]. Therefore, each footwear condition resulted in 24 stance phases for time- and frequency-domain analysis. All parameters from the acceleration signal in this study were calculated using a custom Python program (v3.8, Python Software Foundation, Wilmington, NC, USA).

The power spectrum was analyzed by transforming time-domain signals into the frequency domain using the discrete fast Fourier transform (FFT). A linear trend was removed by subtracting a least-squares best-fit line from the raw data signal [30]. Zero padding was performed at the end of each acceleration data until the total number of data points was 1024, as required by the FFT (a multiple of a power of two). The power of stance phase in the frequency domain (from 0 to the Nyquist frequency) was evaluated by calculating PSD using a rectangular window (Figure 2d,e). Furthermore, powers and frequencies were normalized to 1 Hz bins [31]. A transfer function [30] was employed to evaluate shock attenuation or gain between the distal and proximal tibia in decibels at each frequency interval using the following formula:

(1) $$\mathrm{Transfer}\;\mathrm{Function}\;=10{\;\log}_{10}\left(PSD_{p\_tibia}/PSD_{d\_tibia}\right)$$

where $PSD_{p\_tibia}$ and $PSD_{d\_tibia}$ are the power spectral densities of the proximal and distal tibia. A positive value of the transfer function depicts a gain in signal strength at each frequency, and a negative value indicates the attenuation in signal power as the impact shock transfers from the proximal to the distal tibia (Figure 2f).

Therefore, time-domain parameters analyzed in this study included peak resultant acceleration and time from initial foot contact to peak resultant acceleration, and frequency-domain parameters contained lower (3–8 Hz) and higher (9–20 Hz) frequency PSD and shock attenuation.

### 2.4. Statistical Analysis

Prior to analysis, the Kolmogorov–Smirnov test was used to check the normality of data distribution, whereas homogeneity was assessed using Levene’s test for homogeneity of variances. Greenhouse–Geisser corrected results are reported if data violated Mauchly’s test for sphericity. A one-way repeated measures analysis of variance (ANOVA) was performed to determine the differences in time and frequency domains among MAXs, CONs, and MINs with a significance accepted at *p* < 0.05. The Bonferroni correction was used for the post hoc pairwise comparison with an adjusted significance level of *p* < 0.017. The effect size was evaluated to quantify the magnitude statistically using the partial eta-squared value ($\eta_{p}^{2}$) and classified as small (0.01 < ES ≤ 0.06), medium (0.06 < ES ≤ 0.14), and large (ES > 0.14) [32]. All statistical analyses were conducted using the SPSS v25 (IBM SPSS inc., Chicago, IL, USA) and GraphPad Prism 9.3.0 (San Diego, CA, USA) statistics software.

## 3. Results

**Figure 3:**
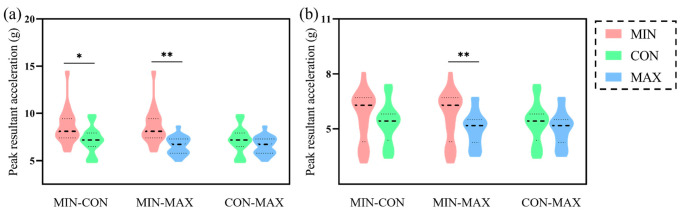
Violin plots of the Bonferroni comparisons for peak resultant acceleration between conditions in the distal (**a**) and proximal (**b**) tibia. Note: MINs: the minimalist shoes; CONs: the conventional shoes; MAXs: the maximalist shoes. * *p* < 0.05 and ** *p* < 0.01. The black dashed line represents the median, and the gray dashed lines above and below represent the third and first quartiles.

No difference was presented for the time to peak acceleration (Table 1). The ANOVA analysis showed that peak resultant accelerations were different statistically among the three footwear conditions in both the distal and proximal tibia (*p* < 0.01, $\eta_{p}^{2}$ = 0.4 and *p* = 0.01, $\eta_{p}^{2}$ = 0.2). Compared with CON and MAX conditions, MINs significantly increased the peak acceleration magnitude of the distal tibia (*p* = 0.01 and *p* < 0.01) (Figure 3a). The peak acceleration in the MIN condition was also greater than the MAXs at the proximal tibia (5.7 ± 1.35 vs. 5.02 ± 0.9 g, *p* < 0.01) (Figure 3b).

**Figure 4:**
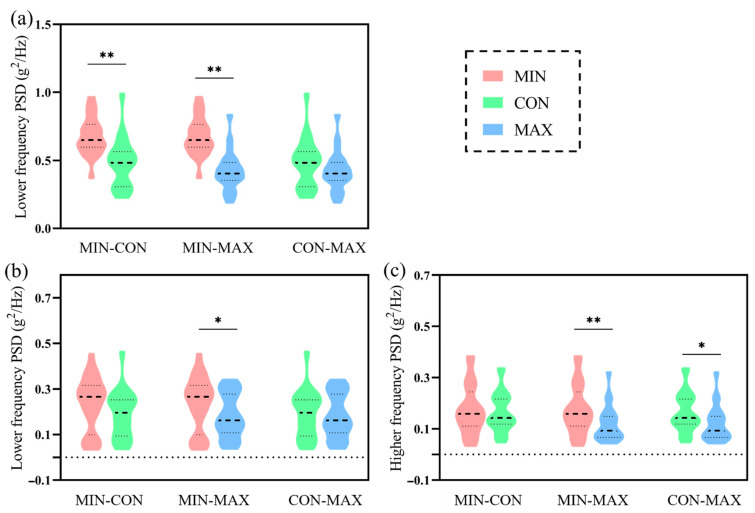
Violin plots of the Bonferroni comparisons for PSD between conditions in the distal (**a**) and proximal (**b**,**c**) tibia. Note: PSD: power spectral density; MINs: the minimalist shoes; CONs: the conventional shoes; MAXs: the maximalist shoes. * *p* < 0.05 and ** *p* < 0.01. The black dashed line represents the median, and the gray dashed lines above and below represent the third and first quartiles.

In the distal tibia, PSD in the lower frequency (3–8 Hz) exhibited statistical differences among the three conditions with $\eta_{p}^{2}$ = 0.45 and *p* < 0.01 (Table 1), and was greater in the MIN condition than the CON (*p* < 0.01) and MAX (*p* < 0.01) conditions (Figure 4a). In the proximal tibia, it was demonstrated that MAXs decreased the PSD in both the lower (*p* = 0.03) and higher (*p* < 0.01) frequency range compared to MINs (Figure 4b,c). PSD in the higher frequency was also less in the statistics than CONs (0.12 ± 0.07 vs. 0.16 ± 0.08 g^2^/Hz, *p* = 0.02). Shock attenuation in the lower frequency depicted no difference but was greater in the MAXs in the higher frequency (9–20 Hz) compared with the MIN condition (−54.72 ± 37.49 vs. −38.27 ± 45.03 dB and *p* < 0.01).

## 4. Discussion

Time- and frequency-domain characteristics were investigated in this study among the MINs, MAXs, and CONs. We found that tibial shock acceleration differs among the different footwear conditions in the time domain and altered PSD and shock attenuation in the frequency dimension. Specifically, MINs increased peak acceleration in the distal tibia, but peak acceleration was not significantly different in the proximal tibia. PSD in the MINs was increased at the distal tibia in the lower frequency. MAXs decreased PSD on the proximal tibia (9–20 Hz). Furthermore, PSD in the MINs was significantly greater than in the MAXs at both the lower and higher frequency ranges in the proximal tibia and the lower frequency range in the distal tibia.

Shock acceleration characteristics in time and frequency dimensions are essential to understand impact loading. Footwear [13] and prolonged running [24] are associated with peak acceleration alterations. The frequency content of the impact loading and how shock acceleration is attenuated are thought to be of greater importance for understanding injury mechanisms and preventing potential injuries than variables in the time domain [21]. Foot strike pattern [21], step length [22], and stride frequency [23] have been previously identified as potential factors that contribute to differences in PSD during running. This study illustrated the differences in the frequency characteristics between MINs and MAXs. Furthermore, MIN running lacks the cushioning function and MAX running decreases impact loading metrics in the time domain, which has been controversial [15,17]. We found that MAXs did not decrease impact acceleration significantly in either the time or frequency domains, except for PSD at the higher frequency in the proximal tibia, which is in contrast with our hypothesis.

Sinclair [19] evaluated shock attenuation and illustrated decreased impact attenuation in the MINs compared to MAXs and CONs. However, it was quantified in the whole lower limb from a time dimension [33]. Shin and knee injuries are predominant in running-related injuries, for instance, patellofemoral pain [34] and bone stress injuries [35]. It is essential to explore the transmission of impact loading from the ankle and distal tibia to the proximal tibia and knee. We could identify that the cushioning function affects the time to peak acceleration, as it was decreased at the proximal tibia in the MINs but increased in the MAXs, although with no significant effect. Therefore, the impact loading rate may be increased in the MINs and decreased in the MAXs as the peak acceleration at the proximal tibia was higher in the MINs, followed by the CONs, which is consistent with the previous finding [36].

The pathomechanical evidence from a previous literature review [35] supports that this enhanced impulse may increase the risk of bone stress injuries in distance runners. The difference in footwear in shock acceleration is present in both the distal and proximal tibia and may be a contributing factor to bone stress fractures. It also appeared that peak acceleration in the MINs was greater than in the CONs at the initial tibia reaction, but it was not significant when the impact acceleration reached the knee, which is consistent with the frequency characteristic in the lower range frequency components. Hence, the cushioning function from footwear in the time domain is more pronounced from the ankle to the distal tibia and dissipates at the proximal tibia. These findings elucidated the shock absorption mechanism at the tibia among different footwear conditions. A previous study from Busa et al. [23] suggested defining the lower frequency signal as the active phase of stance and the higher frequency content as the impact phase; however, impact and active phases were not set to these specific ranges because the Fourier transform loses all time-domain information [30].

Additionally, the PSD difference from the distal to the proximal tibia and shock attenuation characteristics in this study supported that quantifying the shock attenuation at the tibia is critical for understanding acceleration and impact absorption differences among footwear conditions in the time and frequency domains [19,37] because impact loading maintains a similar magnitude from the knee [38].

The greater the shock attenuation, the more impact loading is dissipated by the tibia during the stance phase [21]. In support of our hypothesis, MINs depicted a greater shock attenuation effect in the higher frequency domain than CONs. However, only MAXs showed a significant increase in the shock attenuation. This is inconsistent with our expectation that MINs would exhibit a more considerable shock attenuation than MAXs, compared to the CON condition. It means the cushioning function in the MAXs presented the importance of absorbing shock regarding the frequency content. Therefore, the shock attenuation function in the MAXs exist as the footwear manufacturer claimed, rather than just as a commercial advert. Investigating the time and frequency contents for the MAXs goes beyond understanding the impact loading during running in just the time domain [15,20] and sheds light on the understanding of shock acceleration in the frequency content. These findings have potential clinical implications, specifically for preventing tibial stress injuries in recreational male runners using wearable sensors.

One limitation in this study should be considered, namely, only male habitual rearfoot strike runners were recruited in this study. Therefore, these findings are not suggested to apply for the habitual mid- and forefoot strike runner. Future studies may be interested in investigating shock attenuation characteristics on forefoot strikers in the time and frequency contents between MIN and MAX conditions. Differences between male and female runners may also reveal differences in the injury mechanics in the lower extremities, which is worth exploring in the future.

## 5. Conclusions

This study is a timely addition to the literature regarding the time-domain tibial shock and frequency-domain shock attenuation between the distal and proximal tibia in male recreational runners between MIN, MAX, and CON conditions. We found that peak acceleration and PSD at the distal tibia were significantly greater in the MINs than CONs during running, but the difference disappeared when the impact loading transferred to the proximal tibia. However, MINs demonstrated no significant shock attenuation effect. These findings may provide tibial shock information for choosing running shoes and preventing tibial stress injuries. It is suggested that novice runners and recreational runners with a history of tibial stress fractures do not use the MINs as their preference in daily running.

## Figures and Tables

**Table 1:** Tibial acceleration analysis in the time and frequency domains with different running shoes (data were presented in mean (SD)).

	MINs	CONs	MAXs	One-Way ANOVA		
				F-Value	$\eta_{p}^{2}$	*p*-Value
**Time domain**						
*Distal tibia*						
Time to peak acceleration (s)	0.01 (0.00)	0.01 (0.00)	0.01 (0.01)	1.18	0.05	0.31
Peak resultant acceleration (g)	8.52 (1.75)	7.13 (1.37)	6.58 (0.91)	15.27	0.4	<0.01
*Proximal tibia*						
Time to peak acceleration (s)	0.03 (0.03)	0.05 (0.04)	0.06 (0.06)	2.01	0.08	0.15
Peak resultant acceleration (g)	5.7 (1.35)	5.32 (1.10)	5.02 (0.90)	5.73	0.2	0.01
**Frequency domain**						
*Distal tibia*						
PSD in 3–8 Hz (g^2^/Hz)	0.68 (0.14)	0.48 (0.18)	0.42 (0.14)	18.99	0.45	<0.01
PSD in 9–20 Hz (g^2^/Hz)	0.32 (0.14)	0.26 (0.14)	0.25 (0.10)	2.89	0.11	0.07
*Proximal tibia*						
PSD in 3–8 Hz (g^2^/Hz)	0.23 (0.12)	0.19 (0.10)	0.18 (0.10)	4.30	0.16	0.02
PSD in 9–20 Hz (g^2^/Hz)	0.17 (0.10)	0.16 (0.08)	0.12 (0.07)	6.83	0.23	<0.01
*Shock attenuation*						
3–8 Hz magnitude (dB)	−32.36 (21.28)	−28.12 (23.12)	−24.61 (23.76)	1.98	0.08	0.15
9–20 Hz magnitude (dB)	−38.27 (45.03)	−23.53 (42.64)	−54.72 (37.49)	5.40	0.19	0.01

Note: PSD: power spectral density; MINs: the minimalist shoes; CONs: the conventional shoes; MAXs: the maximalist shoes.

## Data Availability

Data is available on request due to the restriction of ethics.
